# Prevalence and dietary factors associated with nonalcoholic fatty liver disease in a sample of obese middle-aged Egyptian women

**DOI:** 10.1038/s41598-026-42141-7

**Published:** 2026-03-25

**Authors:** Maha I. A. Moaty, Salwa M. El Shebini, Rehab A. Mohamed, Safenaz Y. El Sherity, Nihad H. Ahmed

**Affiliations:** 1 Department of Nutrition and Food Sciences, Food Technology and Nutrition Institute, National Research Centre, 33 El-Buhouth St., Dokki, Giza 12622 Egypt; 2 Department of Medical Biochemistry, Medical Research and Clinical Studies Institute, National Research Centre, 33 El-Buhouth St., Dokki, Giza 12622 Egypt; 3 Department of Biological Anthropology, Medical Research and Clinical Studies Institute, National Research Centre, 33 El-Buhouth St., Dokki, Giza 12622 Egypt

**Keywords:** Nonalcoholic fatty liver disease (NAFLD), Obesity, Dietary habits, Nutritional status, Egyptian women., Biochemistry, Diseases, Health care, Medical research

## Abstract

This study investigated the prevalence of Nonalcoholic Fatty Liver Disease (NAFLD) among a sample of middle-aged obese Egyptian women and explored the associations between nutritional status, dietary habits and NAFLD development, with the aim of increasing nutritional awareness and reducing NAFLD risk. A total of 84 obese women were evaluated using dietary assessments, anthropometric measurements, abdominal ultrasonography, fasting blood glucose, lipid profile and liver function tests. NAFLD was detected in 59% of participants and was associated with obesity, suboptimal dietary habits and low physical activity. Participants with moderate NAFLD had the highest caloric intake, primarily from saturated fats, and exhibited deficiencies in several essential vitamins. NAFLD severity was positively correlated with body mass index, body fat percentage, waist circumference, alanine aminotransferase levels and dietary fat intake, and negatively correlated with most fat-soluble and water-soluble vitamins. In conclusion, these findings suggest that NAFLD is relatively common among obese Egyptian women and is associated with obesity and lifestyle-related factors. Dietary patterns characterized by high energy intake, refined sugars, and unhealthy fats were more frequently observed among participants with greater disease severity. As diet and lifestyle are potentially modifiable risk factors, nutritional education and the promotion of healthier dietary and lifestyle practices may be beneficial in addressing NAFLD risk and progression.

## Introduction

Nonalcoholic fatty liver disease (NAFLD), recently defined as metabolic-associated fatty liver disease (MAFLD)^1^, is characterized by the accumulation of triglycerides (TG)-based fat in the liver, exceeding 5–10% of liver weight, in the absence of significant alcohol consumption. NAFLD has emerged as a critical global public health concern, affecting about one-third of the world’s population^2^. NAFLD involves a wide histological spectrum, ranging from simple steatosis to nonalcoholic steatohepatitis (NASH), hepatic fibrosis, cirrhosis, and hepatocellular carcinoma. In advanced stages, it can progress to end-stage liver disease, necessitating liver transplantation^3^. Therefore, urgent and comprehensive strategies are needed to raise awareness and address this growing health concern on local, regional, and global levels^4^^5^.

Numerous studies have highlighted the strong association between obesity and an increased risk of developing NAFLD, with prevalence rates among obese individuals reported as high as 100%. NAFLD is closely linked to obesity and type 2 diabetes mellitus (T2DM) due to shared features of metabolic syndrome, including chronic inflammation and insulin resistance^6^^7^.

Global epidemiological studies indicate a higher prevalence of NAFLD in higher-income countries, suggesting a link between economic development and disease incidence^8^. On the other hand, the rising prevalence in Eastern and developing nations has been attributed to the increasing consumption of high-energy Western diets, characterized by calorie-dense, nutrient-poor foods, alongside sedentary lifestyles^9^^10^.

Within the Middle East and North Africa (MENA) region, NAFLD affects approximately 31.8% of adults, underscoring the region’s significant disease burden. Many cases of NAFLD remain asymptomatic and undiagnosed, often due to the misconception that affected individuals are healthy. However, the significant prevalence of NAFLD within this population represents a major public health concern, posing a potential burden on healthcare systems in this region^11^.

To date, few studies have evaluated the prevalence of NAFLD among middle-aged individuals in Egypt, which ranks among the top ten nations globally in obesity prevalence, using widely accepted and validated diagnostic techniques. This gap highlights the need for further research employing reliable noninvasive tools, such as transient elastography or ultrasonography, to accurately assess the prevalence and risk factors associated with NAFLD, enabling the development of targeted public health interventions^12^.

The primary objective of this study was to estimate the prevalence of NAFLD among a sample of middle-aged obese Egyptian women and to examine the associations between NAFLD and nutritional status, anthropometric measures and dietary habits. A further objective was to highlight nutritional behavior patterns that may be linked to NAFLD risk, in order to support awareness and early preventive strategies. This study provides preliminary evidence that may guide future longitudinal and interventional research.

## Methodology

### Study design

This cross-sectional study included 84 obese women with a mean age of 52.38 ± 1.12 years (range: 33–68 years), who were enrolled in a weight-loss program at the Nutrition and Food Sciences Department, National Research Centre (NRC), Egypt, between November 2023 and March 2024. All participants were female NRC employees who voluntarily joined the program and met the study’s inclusion criteria.

Following radiological examination using abdominal ultrasonography, the volunteers were classified into three groups; obese without fatty liver, obese with mild fatty liver, and obese with moderate fatty liver.

### Sample size

The sample size was calculated based on prevalence estimation for cross-sectional studies using the standard formula: n= [Z^2^ p (1 − p)]/d^2^, where n is the required sample size, Z is the Z value corresponding to a 95% confidence level (1.96), p is the expected prevalence, and d is the desired margin of error. Assuming an expected prevalence of non-alcoholic fatty liver disease (NAFLD) of 25–30% based on previous regional data, a 95% confidence level, and an acceptable margin of error of 11%, the minimum required sample size ranged from 72 to 81 participants. In addition, based on the observed NAFLD prevalence of approximately 60% in the present study, the minimum required sample size was calculated as 74 participants. The final sample of 84 obese women therefore exceeded these requirements, providing adequate precision for estimating NAFLD prevalence in the study population.

### Ethical approval

The study protocol was reviewed and approved by the Ethical Committee of the National Research Centre (NRC) of Egypt in accordance with the ethical principles of the Declaration of Helsinki (Approval No. 13050204-1). A written informed consent was obtained from each participant after a clear explanation of the study’s objectives.

### Inclusion criteria

Participants were eligible if they were middle-aged obese women who had maintained a stable body weight (± 3 kg) over the preceding three months. All participants were clinically stable and capable of undergoing anthropometric measurements, dietary assessment, and abdominal ultrasonography, as well as providing fasting blood samples. They were willing to provide written informed consents.

### Exclusion criteria

Women with secondary causes of fatty liver, such as alcohol consumption above 20 g/day, chronic viral hepatitis (HBV or HCV), autoimmune liver disease, drug-induced liver injury, hepatic fibrosis or cirrhosis, biliary disease or jaundice due to any other cause. Pregnant or lactating women were also excluded.

All volunteers were subjected to the following:

- Full medical history including important symptoms of liver disease, in addition to clinical examination.
  Symptoms and signs of liver affection included: Discomfort or pain in the epigastric or right upper quadrant of the abdomen, loss of appetite, dyspepsia, heartburn, nausea and vomiting. Skin manifestations included redness of the palms, dark, uneven and heterogeneous spots, particularly on the neck and underarms, as well as yellowish discoloration of the skin and eyes, accompanied by dark urine. Psychological symptoms included lack of concentration, mood swings, depression, and anxiety. Additional issues reported were headaches, weakness, fatigue, and lack of energy^13^.
- *Blood pressure* was measured using a cuff sphygmomanometer while the participants sat quietly. The average of three readings was recorded.
- *Relevant anthropometric measurements* were reported as follows: Body weight and height were recorded with participants standing, wearing minimal clothing, and no shoes. Body mass index (BMI) was calculated as weight (in kilograms) divided by height squared (in meters). Minimal waist circumference (MWC) was measured in centimeters using a non-extendable tape during normal respiration. Waist-to-height ratio (WHtR), an indicator of visceral obesity was calculated^14^. Body fat percentage (%BF) was measured as a percentage of body weight using the Geratherm Body Fitness device (B-5010). All measurements were taken by an expert clinician to ensure accuracy.
- *Dietary assessment*: Dietary intake was estimated using three successive 24-hour recalls, including one on a weekend day, to account for variations in the dietary pattern, in combination with a semi-quantitative FFQ adapted to Egyptian eating habits. The portion sizes were estimated using standardized food models provided, along with household measures specified in the Egypt National Nutrition Survey. The nutrient intake was analyzed using NutriSurvey 2007, customized with Egyptian food composition data to enhance cultural and nutritional relevance. Previous studies^15–17^ supported the reliability of this software in Egyptian populations, which included national food databases and decomposed mixed dishes into ingredients for accurate nutrient estimation. This enhances the validity and sufficiency of our method of dietary assessment.
- *Blood sampling and biochemical analysis*: Blood samples were collected on the day of clinical examination following an overnight fast. Fasting blood glucose levels were measured immediately from fresh samples using test strip electrochemical technology with a calibrated Bayer’s CONTOUR® PLUS glucometer^18^, validated for accuracy and reliability in both clinical and field settings. This method was selected for its feasibility, participant convenience, and suitability for community-based data collection. The remaining blood samples were collected in plain red-top tubes, allowed to clot at room temperature, and then centrifuged. The sera were separated and divided into aliquots in Eppendorf tubes. Additional biochemical analyses were conducted on fasting sera, which were stored at −70 °C until use.
  Lipid profile parameters including total cholesterol (TC), high-density lipoprotein cholesterol (HDL-C), and triglycerides (TG) levels were measured using enzymatic methods with kits provided by Erba Lachema s.r.o., Karásek 1 d, 621 00 Brno, CZ^19^.
  Liver function tests, including liver enzymes: Alanine aminotransferase (ALT), aspartate aminotransferase (AST) and gamma-glutamyl transferase (GGT), in addition to total protein and total bilirubin, were evaluated using colorimetric methods^20^.
- *Radiological examination of the liver* was performed using abdominal ultrasonography^21^, a noninvasive diagnostic tool, on a separate day after a 6-hour fast to reduce bowel gas interference and ensure optimal liver visualization. An experienced sonographer who was unaware of any clinical information about the examined person did the procedure. Initially, the examination was conducted using the GE HD Voluson P8 system. This first assessment allowed detection of fatty liver; however, due to the limitations of the Voluson P8 in providing precise grading of hepatic steatosis, further evaluation was required. Subsequently, a more specialized assessment was performed using the Siemens ACUSON S3000 Ultrasound-HFLX system equipped with a 6C1HD curved array transducer and Acoustic Radiation Force Impulse Imaging (ARFI) technology (Virtual Touch Imaging and Virtual Touch Quantification). This approach enabled both qualitative and quantitative analysis of liver stiffness, allowing accurate grading of fatty liver. The procedure measured liver stiffness at seven hepatic segments. Data included in this research focused only on steatosis findings (fatty liver), while other conditions such as coarse parenchyma, fibrosis, fat-sparing areas or focal lesions were excluded to concentrate on fatty liver staging.

The grading system for fatty liver echogenicity was as follows^22^:

- Grade 0: Absence of steatosis (fat content < 5% of liver weight) with normal liver echogenicity, indicating no fatty liver disease.
- Grade 1: Mild steatosis (fat content 5–33%), with liver echogenicity higher than the right renal cortex but preserved echogenic wall of the main portal vein, indicating mild fatty liver disease.
- Grade 2: Moderate steatosis (fat content 34–66%), with impaired echogenicity of the main portal vein wall, indicating moderate fatty liver disease.
- Grade 3: Severe steatosis (fat content > 66%), with impaired visualization of the posterior hepatic parenchyma or diaphragm, indicating severe fatty liver disease.

### Statistical analysis

Statistical analyses were performed using SPSS software (version 26.0; IBM Corp., Chicago, IL, USA). Data normality and homogeneity of variances were assessed using the Kolmogorov-Smirnov and Levene’s tests, respectively. As assumptions were met, parametric tests were applied, and results were presented as mean ± SEM. Group differences were analyzed using one-way ANOVA with post hoc multiple comparisons and associations between continuous variables were assessed using Pearson’s correlation coefficient. Odds ratios and their 95% confidence intervals were calculated for the risk factors in relation to NAFLD severity. A two-tailed p-value < 0.05 was considered statistically significant.

## Results

**Fig. 1:**
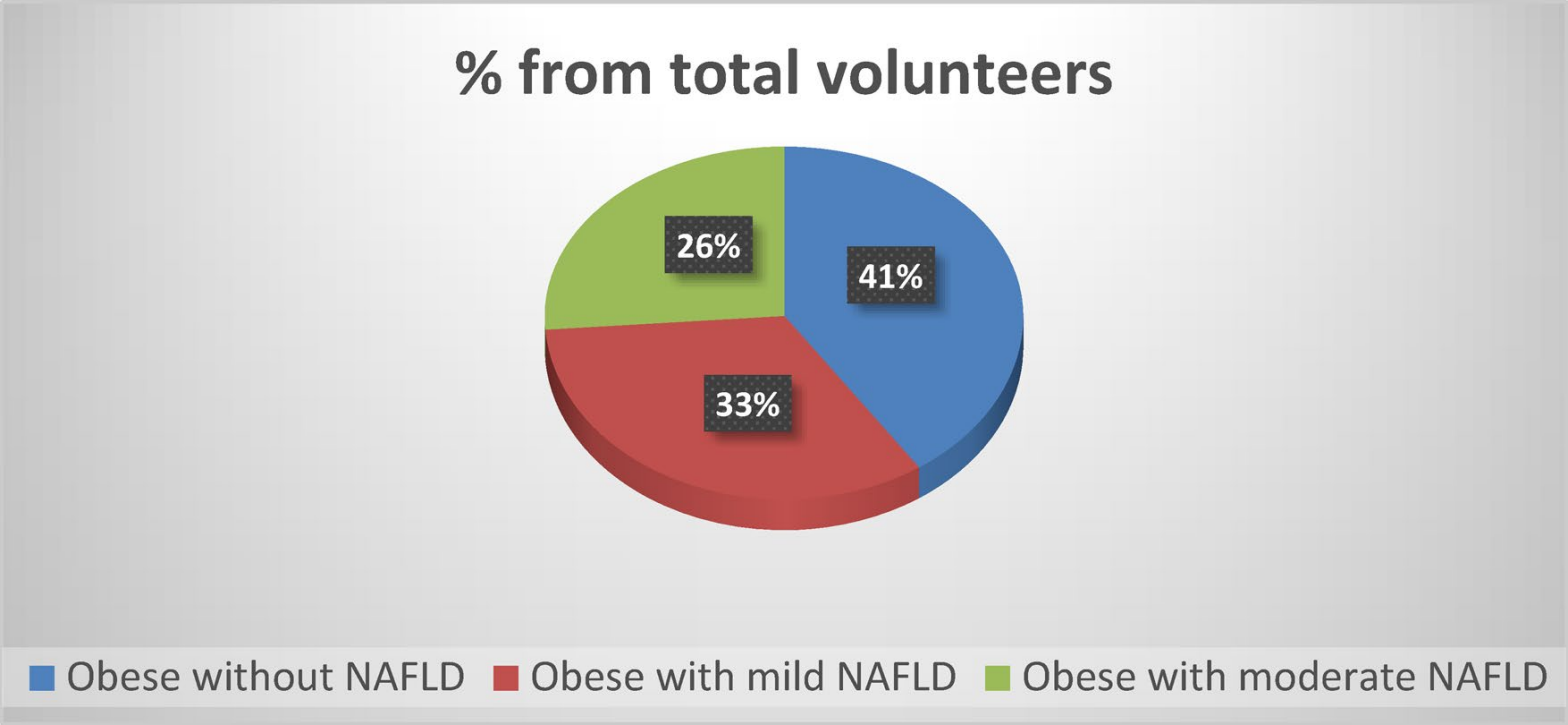
Classification of obese subjects as a percentage from total volunteers according to presence or absence of fatty liver disease and its degree of severity.

After radiological examinations, by abdominal ultrasonography, about 59% (50 subjects) of volunteers were detected to have fatty liver disease. The NAFLD subjects were classified according to the severity of fatty liver disease to mild NAFLD group (Grade 1) which represents about 33% (28 subjects) of the total volunteers, and a moderate NAFLD group (Grade 2) that represents about 26% (22 subjects) of the total volunteers (Fig. 1).

**Table 1:** Comparison of the risk factors in obese women with and without NAFLD.

Parameters	Obese without NAFLD (Grade 0: no.=34)		Obese with NAFLD (Grade 1& 2: no.=50)		Prevalence ratio	OR (95% CI)	*P*-value
	No.	%	No.	%			
Presence of hepatic symptoms	18	52.9	36	72.0	1.36	2.29 (0.92–5.70)	0.065
History of renal affection	2	5.9	10	20	3.39	4.00 (0.82–19.57)	0.073
History of hypertension	10	29.4	31	62.0	2.11	3.92 (**1.54–9.95)**	**0.005**
History of diabetes mellitus	8	23.5	14	28.0	1.19	1.26 (0.46–3.45)	0.660
Obesity (BMI ≥ 30 kg/m^2^)	26	76.5	48	96	1.25	7.38 (**1.46–37.37)**	**0.022**
Waist to height ratio ≥ 0.5	32	94.1	50	100	1.06	not calculable*	0.134
Overconsumption of calorie rich foods	20	58.8	44	88	1.50	5.13 (**1.72–15.31)**	**0.007**
Sedentary life & lack of physical activity	25	73.6	48	96	1.30	8.64 (**1.73–43.08)**	**0.013**

NAFLD: Nonalcoholic Fatty Liver Disease. Data are presented as number (No.) and percentage (%). Odds ratios (ORs) with 95% confidence intervals (CIs) were calculated using univariate binary logistic regression (Wald method). Categorical variables were compared using the chi-square or Fisher’s exact test, as appropriate. A p-value < 0.05 was considered statistically significant. *The OR for waist-to-height ratio was not calculable due to complete exposure among participants with NAFLD (100%).

Table 1 showed that modifiable lifestyle factors were the strongest predictors of NAFLD in this study. A sedentary lifestyle was most strongly associated with NAFLD (OR = 8.64, 95% CI: 1.73–43.08), followed by general obesity (BMI ≥ 30 kg/m²) (OR = 7.38, 95% CI: 1.46–37.37) and high consumption of calorie-rich foods as food high in refined carbohydrate and saturated fat (OR = 5.13, 95% CI: 1.72–15.31), highlighting the impact of physical inactivity and dietary habits on liver health. Central obesity, assessed by waist-to-height ratio (WHtR ≥ 0.5), was present in 100% of participants with NAFLD, reflecting its critical role in disease development. Among metabolic comorbidities, only hypertension showed a significant association with NAFLD (OR = 3.92, 95% CI: 1.54–9.95). Notably, 28% of participants with NAFLD were asymptomatic at assessment, emphasizing the importance of proactive screening even in the absence of clinical symptoms. These findings underscore the relevance of targeting modifiable lifestyle factors in the prevention and management of NAFLD.

**Table 2:** Comparison between the characters of the studied groups regarding age, anthropometric measurements, blood pressure and some relevant biochemical markers.

Parameters	Obese without NAFLD (Grade 0: no.=34)	Obese with mild NAFLD (Grade 1: no.=28)	Obese with moderate NAFLD (Grade 2: no.=22)	Total (no.=84)
	Mean ± SE			
Age (years)	46.06 ± 1.75 a	52.91 ± 2.12 b	59.17 ± 1.04 c	52.38 ± 1.12
Weight (kg)	84.14 ± 2.59 a	86.36 ± 2.89 a	95.25 ± 2.59 b	87.76 ± 1.64
Height (cm)	156.12 ± 1.12 a	156.00 ± 1.26 a	156.64 ± 1.03 a	156.21 ± 0.67
BMI (kg/m^2^**)**	34.50 ± 0.96 a	35.23 ± 0.78 a	38.87 ± 1.05 b	35.87 ± 0.58
WC (cm)	90.06 ± 1.93 a	89.43 ± 1.54 a	95.82 ± 1.52 b	91.31 ± 1.04
WHtR (cm/cm)	0.58 ± 0.01 a	0.57 ± 0.01 a	0.61 ± 0.01 b	0.58 ± 0.01
Body fat (%)	45.51 ± 0.87 a	47.13 ± 0.61 a	49.61 ± 0.75 b	47.13 ± 0.48
SBP (mmHg)	124.65 ± 2.76 a	128.20 ± 3.56 a	128.55 ± 2.72 a	126.88 ± 1.78
DBP (mmHg)	79.12 ± 1.84 a	77.20 ± 1.79 a	81.82 ± 1.96 a	79.14 ± 1.09
FBG (mg/dL)	101.80 ± 2.63 a	109.91 ± 5.08 a	117.47 ± 8.45 a	110.07 ± 3.74
TC (mg/dL)	258.17 ± 2.92 a	273.33 ± 7.07 b	276.52 ± 3.64 b	268.32 ± 3.04
TG (mg/dL)	116.64 ± 4.93 a	117.53 ± 3.14 a	122.60 ± 5.13 a	118.51 ± 2.49
HDL-C (mg/dL)	49.21 ± 1.56 a	47.73 ± 1.01 a, b	44.50 ± 1.25 b	47.44 ± 0.77
Non-HDL-C (mg/dL)	208.96 ± 3.65 a	225.60 ± 3.57 a, b	232.02 ± 8.23 b	220.88 ± 3.49

BMI: Body mass index, WC: Waist circumference, WHtR: Waist to height ratio, SBP: Systolic blood pressure, DBP: Diastolic blood pressure, FBG: Fasting blood glucose, TC: Total cholesterol, TG: Triglycerides, HDL-C: High density lipoprotein cholesterol. Groups sharing the same initial at the same row were not statically significant.

Table 2 showed the characters of the studied groups regarding their age, anthropometric measurements, blood pressure, and some relevant biochemical markers. Data revealed that both the NAFLD groups were older than the healthy liver group. Significant differences were recorded among the three groups regarding the age, weight, BMI, WC, WHtR, % body fat, TC, HDL-C and non-HDL-C especially between the healthy liver group and the moderate NAFLD group at the level of *P* < 0.05 − 0.01. The mean values of fasting blood glucose and blood pressure were within normal ranges as the hypertensive and diabetic patients were under treatment.

**Table 3:** Comparison between the studied groups regarding their biochemical liver profile.

Parameters	Obese without NAFLD (Grade 0) no.=34)	Obese with mild NAFLD (Grade 1 no.=28)	Obese with moderate NAFLD (Grade 2 no.=22)	Total (no.=84)
	Mean ± SE			
ALT (U/L)	12.80 ± 0.96 a	16.73 ± 1.02 b	20.57 ± 1.27 c	17.10 ± 0.73
AST (U/L)	19.87 ± 0.94 a	20.14 ± 1.09 a	20.60 ± 1.11 a	20.154 ± 0.59
GGT (U/L)	14.93 ± 0.17 a	15.87 ± 0.35 a	16.11 ± 0.28 a	15.72 ± 0.18
T. Protein (g/dL)	6.91 ± 0.07 a	7.05 ± 0.04 a	7.03 ± 0.11 a	6.99 ± 0.04
T. Bilirubin (mg/dL)	0.59 ± 0.02 a	0.64 ± 0.02 a, b	0.67 ± 0.02 b	0.63 ± 0.01

ALT: Alanine aminotransferase, AST: Aspartate aminotransferase, GGT: Gamma-glutamyl transferase. Groups sharing the same initial at the same row were not statically significant.

Table 3 provided a comparative analysis of the biochemical liver profile across the three studied groups. Significant differences were detected in ALT levels among all groups, with a notable increase in the moderate NAFLD. Additionally, total bilirubin level was significantly elevated in this group compared to the healthy liver group. Other liver function parameters, such as AST and GGT, showed a numerical increase in their mean values in the moderate NAFLD group compared to the other groups, although these differences did not reach statistical significance.

Tables 4 and 5 illustrated the mean daily macro and micronutrients’ intake, and their percentage relative to the Recommended Dietary Allowances (RDAs) among the three obese groups. The values of RDA were defined according to World Health Organization (WHO)/Food and Agriculture Organization of the United Nations (FAO)^23^. The findings revealed high consumption of energy, protein, fat and saturated fat across all the three groups. Relative to the RDAs, the Moderate NAFLD group demonstrated the highest percentages daily intake of calories and macronutrients compared to the other two groups, with significant differences noted. The calories consumed by the Moderate NAFLD group primarily came from fat (represents about 160.23% of the RDAs), characterized by high saturated fat and cholesterol contents and low levels of monounsaturated fatty acids (MUFAs) and polyunsaturated fatty acids (PUFAs). Additionally, this group showed deficiencies in the dietary fiber, vitamin A, vitamin D, vitamin E, vitamin K and the B vitamins: B1, B2, B6, and B12, niacin, pantothenic acid and folic acid, in addition to vitamin C, and selenium. In contrast, sodium intake was elevated compared to the RDAs, with significant differences observed.

**Table 4:** Mean ± SE of macronutrients intake and their percent from the RDA for the studied groups.

Nutrient intake	Obese without NAFLD (Grade 0)	Obese with mild NAFLD (Grade 1)	Obese with moderate NAFLD (Grade 2)	RDA
	Mean ± SE %RDAs			
Energy (kcal)	2037.83 ± 33.96 a 101.89%	2159.23 ± 45.67 b 107.96%	2288.82 ± 26.80 c 114.44%	2000
Protein (g)	69.72 ± 4.95 a 139.44%	74.23 ± 4.67 a 148.46%	79.46 ± 8.06 a 158.92%	50
Fat (g)	88.42 ± 5.07 a 136.03%	93.02 ± 6.45 a 143.11%	104.15 ± 6.38 b 160.23%	65
SFAs (g)	35.44 ± 1.31 a 15.95%	41.63 ± 0.81 b 18.73%	45.95 ± 0.86 c 20.68%	No more than 7% Total Calories
MUFAs (g)	8.48 ± 0.41 a 3.82%	6.25 ± 0.38 b 2.81%	4.29 ± 0.32 c 1.93%	12%−14% Total Calories
PUFAs (g)	6.23 ± 0.24 a 2.80%	4.40 ± 0.35 b 1.98%	3.29 ± 0.26 c 1.48%	6%−8% Total Calories
Cholesterol (mg)	143.15 ± 6.24 a 71.56%	151.13 ± 1.97 a 75.57%	217.72 ± 3.49 b 107.78%	200
Carbohydrates (g)	240.96 ± 10.24 a 80.32%	250.03 ± 13.61 a 83.35%	251.85 ± 15.20 a 83.95%	300
Dietary fiber (g)	18.42 ± 0.29 a 73.68%	16.65 ± 0.44 b 66.60%	12.87 ± 0.42 c 51.48%	25

SFAs: Saturated fatty acids, MUFAs: Monounsaturated fatty acids, PUFAs: Polyunsaturated fatty acids.

Groups sharing the same initial at the same row were not statically significant.

**Table 5:** Daily micronutrient intake (mean ± SE) and their percent of recommended daily allowance (%RDA) based on updated DRI (WHO/FAO) values for the studied groups.

Nutrient intake	Obese without NAFLD (Grade 0)	Obese with mild NAFLD (Grade 1)	Obese with moderate NAFLD (Grade 2)	RDA
	Mean ± SE %RDAs			
Vitamin A (µg)	531.26 ± 15.06 a 66.41%	429.34 ± 31.90 b 53.67%	320.52 ± 20.34 c 40.07%	800
Vitamin D (µg)	3.45 ± 0.15 a 23.00%	3.17±0.018 a, b 21.33%	2.68 ± 0.20 b 17.87%	15
Vitamin E (mg)	3.43 ± 0.21 a 28.33%	3.23 ± 0.12 a, b 26.92%	2.86 ± 0.14 b 23.83%	12
Vitamin K (µg)	37.52 ± 1.06 a 46.90%	30.22 ± 1.15 b 37.78%	24.40 ± 1.33 b 30.50%	80
Vitamin B1 (yhiamin) (mg)	0.79 ± 0.26 a 71.82%	0.71 ± 0.35 b 64.55%	0.69 ± 0.12 b 62.73%	1.1
VitaminB2 (riboflavin) (mg)	0.80 ± 0.09 a 66.67%	0.73 ± 0.03 b 60.83%	0.70 ± 0.01 b 58.33%	1.2
VitaminB6 (mg)	0.73 ± 0.02 a 56.15%	0.71 ± 0.02 a 54.62%	0.66 ± 0.01 b 50.77%	1.3
Niacin (mg)	7.57 ± 0.20 a 57.35%	7.13 ± 0.27 a, b 54.02%	6.83 ± 0.14 b 51.74%	13.2
Pantothenic acid (mg)	3.80 ± 0.09 a 76.00%	3.28 ± 0.12 b 65.60%	2.70 ± 0.08 c 54.00%	5
Folate (DFE)	240.13 ± 1.03 a 60.03%	218.48 ± 1.05 b 54.62%	205.88 ± 1.07 b 51.47%	400
VitaminB12 (µg)	1.07 ± 0.19 a 53.50%	0.94 ± 0.08 b 47.00%	0.74 ± 0.11 c 37.00%	2
Vitamin C (mg)	35.92 ± 0.55 a 59.87%	28.75 ± 1.39 b 47.92%	24.97 ± 0.49 c 41.62%	60
Sodium (mg)	1338.43 ± 13.40 a 89.23%	1439.22 ± 18.29 b 95.95%	1461.61 ± 11.95 b 97.44%	1500
Potassium (mg)	1330.32 ± 14.88 a 66.52%	1339.63 ± 15.33 a 66.98%	1402.91 ± 22.58 b 70.15%	2000
Selenium (µg)	39.23 ± 0.96 a 71.32%	33.23 ± 0.37 b 60.42%	31.22 ± 0.40 c 56.76%	55

Groups sharing the same initial at the same row were not statically significant.

RDA (Recommended Daily Allowance). Source: WHO/FAO^**23**^.

**Table 6:** Comparing the percent of the subjects regarding the frequency consumption of different food groups in the studied groups.

Food items	Obese without NAFLD (Grade 0)					Obese with mild NAFLD (Grade 1)					Obese with moderate NAFLD (Grade 2)				
	Every day	Twice/week	Every week	Less	Never or rarely ever	Every day	Twice/week	Every week	Less	Never or rarely ever	Every day	Twice/week	Every week	Less	Never or rarely ever
Carbohydrate foods (bread, bakery products, pasta, etc.)	29.41	25.49	19.61	19.61	5.88	40.48	35.71	16.67	4.76	2.38	48.49	30.30	21.21	0.00	0.00
Sig 2-tailed	a					a, b					b				
Milk & milk products (milk, cheese, yoghurt, etc.)	19.61	29.41	25.49	17.65	7.85	16.66	23.81	26.19	19.05	14.29	6.06	15.15	30.30	27.28	21.21
Sig 2-tailed	a					a, b					b				
Animal protein foods (chicken, meat, fish, egg, etc.)	10.29	19.12	27.94	23.53	19.12	10.72	26.78	26.79	21.43	14.28	11.36	20.46	29.55	20.45	18.18
Sig 2-tailed	a					a					a				
Plant protein food (legumes, beans, pulses, etc.)	52.94	29.41	17.65	0.00	0.00	42.86	35.71	7.14	14.29	0.00	45.45	36.36	9.10	9.09	0.00
Sig 2-tailed	a					a, b					b				
Vegetables (fresh vegetables& cooked vegetable)	52.95	20.59	23.53	2.93	0.00	24.99	32.14	17.86	14.29	10.72	13.64	22.73	40.92	22.71	0.00
Sig 2-tailed	a					a, b					b				
Fruits (fresh fruits, fruit juices, etc.)	17.65	32.35	29.41	14.71	5.88	10.72	25.00	17.86	39.28	7.14	9.09	18.18	27.27	36.37	9.09
Sig 2-tailed	a					a, b					b				
Sweets, Snacks high in sugar, salt and fat.	17.65	29.41	35.30	5.88	11.76	28.57	42.86	21.43	7.14	0.00	36.37	45.45	18.18	0.00	0.00
Sig 2-tailed	a					a, b					b				
Sugar sweetened beverages (tea, carbonated drink, etc.)	26.47	26.47	32.35	2.94	11.77	32.15	32.14	21.43	3.56	10.72	40.90	27.28	9.09	13.64	9.09
Sig 2-tailed	a					a, b					b				

Groups sharing the same initial at the same row were not statically significant.

Table 6 represented the percentage of the subjects in the studied groups regarding the frequency consumption of different food groups. The findings revealed that carbohydrate-based food (bread, bakery products and pasta) as well as the plant protein foods (beans and legumes) were the most frequent daily consumed items among obese with NAFLD groups (40.48%& 42.86% of mild NAFLD and 48.49%& 45.45% of moderate NAFLD respectively). Milk and dairy product consumption decreased in the two NAFLD groups. The frequency of vegetable and fruit consumption was low among both the NAFLD groups, while it was notably higher in the obese without NAFLD (52.95& 17.65% respectively). In contrast, the frequency of animal protein foods (chicken, meat, fish and egg) was very low among the three groups. Additionally, the frequency of consuming sweets, junk foods and sugar sweetened beverages was highest among the obese with moderate NAFLD (36.37%& 40.90 respectively). Significant differences were observed between obese without NAFLD group and the moderate NAFLD group regarding the frequency of consumption of carbohydrate-based food, milk and dairy products, vegetables and fruits, sweet and Junk foods and sugar sweetened beverages.

**Table 7:** Correlations coefficient between some important anthropometric measurements and dietary items (total fat, fatty Acids and vitamins) and the score of fatty liver disease.

Parameters	Obese without NAFLD (Grade 0: no.=34)		Obese with mild NAFLD (Grade 1: no.=28		Obese with moderate NAFLD (Grade 2: no.=22)	
	*r*	*p*	*r*	*p*	*r*	*p*
BMI (kg/m^2^)	N.S.		N.S.		0.430**	0.003
% Body fat	− 0.397**	0.001	N.S.		0.347*	0.026
Waist (cm)	N.S.		N.S.		0.522**	0.001
WHtR (cm/cm)	N.S.		N.S.		0.563**	0.001
ALT (U/L)	N.S.		N.S.		0.664**	0.001
Total dietary fat (g)	− 0.387**	0.001	0.279*	0.048	N.S.	
Cholesterol (mg)	0.419**	0.001	0.469**	0.001	0.473**	0.001
SFAs (g)	N.S.		0.398**	0.004	0.590**	0.001
MUFAs (g)	0.484**	0.001	− 0.317*	0.023	− 0.683**	0.001
PUFAs (g)	0.334**	0.004	N.S.		N.S.	
Vitamin A (µg)	0.711**	0.001	− 0.781**	. −001	− 0.337*	0.031
Vitamin D (µg)	N.S.		− 0.422**	0.002	− 0.292*	0.050
Vitamin E (mg)	0.429**	0.001	N.S.		− 0.341*	0.028
Vitamin K (µg)	N.S.		N.S.		N.S.	
VitaminB6 (mg)	0.625**	0.001	− 0.268*	0.036	− 0.514**	0.001
Folic acid (DFE)	N.S.		N.S.		− 0.510**	0.001
VitaminB12 (µg)	N.S.		NS		− 0.545**	0.001
Vitamin C (mg)	0.211*	0.050	− 0.254*	0.050	N.S.	

WHtR: Waist to Height Ratio SFAs: Saturated Fatty Acids, MUFAs: Monounsaturated Fatty Acids, PUFAs: Polyunsaturated Fatty Acids.

*Correlation is significant at the 0.05 level; **Correlation is significant at the 0.01 level.

Correlations coefficient between some important anthropometric measurements, dietary items and the score of fatty liver disease was shown in Table 7. The study revealed significant positive correlations between fatty liver severity (Grade 2 NAFLD) and various anthropometric (BMI, % Body fat, Waist, WHtR) and biochemical (ALT) factors. Furthermore, a significant positive correlation was detected between NAFLD score (Grade 1 and Grade 2) and total dietary fat, saturated fatty acids and cholesterol which was more prominent as the degree of fatty liver gets more worsen. On the other hand, negative correlations were detected between the degree of fatty liver and the vitamins (A, D, E, B6, B12, folic acid and C), and MUFA. Controversially, positive correlation was detected between the healthy liver (Grade 0), and the UFAs (MUFAs& PUFAs) and the fat-soluble vitamins (A& E), vitamin B6 and vitamin C.

## Discussion

In Egypt, liver diseases especially cirrhosis and cancer are the 2nd most common cause of death after the cardiovascular diseases. Liver fibrosis represents a serious chronic health problem to Egyptian population reflecting its bad impact on Egyptian economy. Limited literature has examined the epidemiology of non-alcoholic fatty liver disease (NAFLD) among adults in Egypt, a country with one of the highest obesity rates globally^24^.

To the best of our knowledge, this study represents one of the few studies that attempt to evaluate the prevalence of NAFLD among Egyptian middle-aged women (their mean age was 52.38 years ranged from 33 to 68 years), using ultrasonography. This approach provides a non-invasive and reliable assessment of hepatic steatosis and fibrosis, offering valuable insights into the epidemiology and severity of NAFLD in this population. Our estimated prevalence rate of NAFLD among the studied sample was about 59% (50 subjects) as diagnosed and graded by radiological examination performed by an expert doctor. About 33% (28 subjects) of the total sample had mild NAFLD (Grade 1), while about 26% (22 subjects) of all the volunteers had moderate NAFLD (Grade 2) as shown in Fig. 1.

In a previous cohort study, the prevalence rate of NAFLD among 132 Egyptian young adults’ college students was 31.6%^25^, which is exactly similar to the prevalence rate among 1592 adults’ general Middle Eastern populations (31.8%) that was shown in a meta-analysis of several epidemiological studies. The high prevalence of NAFLD detected in the present study could be attributed to obesity. Despite the rising prevalence of NAFLD, a recent study revealed that Egyptians exhibit low to moderate knowledge regarding fatty liver, including its risk factors, preventive strategies and available therapies. However, a notable misconception was prevalent among all respondents-that NAFLD is purely a familial disease and exclusively affects older individuals. To address this issue, a fundamental shift in healthcare management is essential. This approach should give the priority to prevention, through a proactive lifestyle intervention, and the early detection and management of NAFLD^26^.

The finding of this research indicated that the two NAFLD groups were older than the healthy liver group, with significant differences among the three groups. These results were in accordance with the results obtained by Lin et al.^27^ who stated that NAFLD prevalence increases with age. Another study stated that the prevalence rate of NAFLD rising from 5.8% in the people aged < 45 years to 10.3% in people aged ≥ 45 years old^28^. Age-related reductions in liver function have been linked to physiological changes such as decreased hepatic blood flow and a reduction in liver volume; both of them are prominent with advancing age^29^.

Data of the present study denoted that 96% of the two NAFLD groups were obese (BMI ≥ 30 kg/m^2^), furthermore, all the NAFLD subjects had visceral obesity as indicated by waist to height ratio (WHtR) ≥ 0.5. A notable positive correlation was identified between BMI and percentage of body fat with the NAFLD risk in patients with moderate severity. Additionally, a highly significant correlation was observed with waist circumference. Peng et al.^30^ stated that obesity, particularly visceral obesity, is a significant specific risk factor for NAFLD development. Ectopic fat accumulation, including visceral fat and liver fat, predispose to adipose tissue dysfunction, which in turn interfere with the production of adipocytokines, which are essential regulators of metabolic processes. As a result, there is a shift toward increased production of pro-inflammatory cytokines, such as tumor necrosis factor-alpha (TNF-α) and interleukin-6 (IL-6). This pro-inflammatory state promotes systemic inflammation, insulin resistance, and hepatic lipid accumulation, thereby accelerating the progression of NAFLD from simple steatosis to more severe forms, such as non-alcoholic steatohepatitis (NASH) and fibrosis^31^.

Nutrition and dietary habits are the basic corners for the development, progression, and prevention of NAFLD. The interaction between diet composition, nutrient quality, and excess calorie significantly influences hepatic fat accumulation, insulin resistance, and systemic inflammation^32^. The data of this study revealed that 88% of all the NAFLD subjects overconsumed calorie-rich foods. This result highlights the unhealthy dietary habits as a major risk factor for the disease development. Additionally, 96% of these individuals reported a sedentary lifestyle with lack of physical activity, further exacerbating their risk for NAFLD development and progression.

Chronic caloric excess, regardless of macronutrient source, predisposes individuals to obesity, insulin resistance, and NAFLD. Overeating, combined with sedentary behavior, leads to positive energy balance, with excess energy being stored as hepatic fat^32^. The calories consumed by the Moderate NAFLD group primarily came from fat (represents about 160.23% of the RDAs), characterized by high saturated fat content and low levels of MUFAs and PUFAs. Additionally, a significant positive correlation between the degree of fatty liver and the total dietary fat intake, SFAs and cholesterol, while a high significant negative correlation with MUFAs and the fatty liver score were detected. It has been reported that diets high in SFAs and trans fats contribute significantly to hepatic steatosis by increasing lipid deposition and impairing mitochondrial function. These types of fats are prevalent in the Western dietary pattern as processed foods, fried items and red meats. Conversely, diets deficient in MUFAs and omega-3 PUFAs, known for their anti-inflammatory and lipid-regulating properties, further aggravate the imbalance^33^.

The frequency of daily consumption of snacks high in sugar, and sugar sweetened beverages was highest among the obese with moderate NAFLD (36.37%& 40.90 respectively). Significant differences were observed between obese without NAFLD group and the moderate NAFLD group regarding the frequency of consumption of carbohydrate-based food, milk and dairy products, sweet, junk foods and sugar sweetened beverages. Witek et al.^34^ reported that diets rich in simple sugars, such as fructose, sucrose and glucose, are strongly implicated in NAFLD pathogenesis. Fructose, commonly found in sweetened beverages, snacks and processed foods, is particularly lipogenic. Excessive fructose consumption increases lipogenesis, triglyceride accumulation and oxidative stress in the liver. Moreover, it promotes insulin resistance and systemic inflammation, exacerbating hepatic steatosis and fibrosis. Lower level of the dietary fiber intake was reported in the moderate NAFLD group (about 51.5% of the RDA). Fiber plays a protective role in liver health by modulating postprandial glycemia, reducing lipid absorption and supporting a healthy gut microbiome. A low-fiber diet, often associated with low consumption of fruits, vegetables, legumes and whole grains, predisposes to metabolic syndrome and hepatic steatosis^35^.

The current study’s data showed a high shortage of the majority of dietary vitamins, including fat and water-soluble vitamins, which is indicative of their inadequate intake of food sources. This shortage was obvious among the patients suffering from mild and moderate NAFLD. A lack of antioxidant nutrients, such as vitamins E, D, and C, along with polyphenols, may worsen NAFLD by increasing oxidative stress and inflammation, which are key factors in disease progression^36^. The findings of the current study indicated a significant negative correlation between fatty liver disease scores and the intake of dietary vitamins A, D, and E. Conversely, research conducted by Tang et al.^37^ did not establish a direct relationship between total dietary vitamin A consumption and the risk of NAFLD after accounting for confounding variables.

Lower level of Vitamin D is frequently observed in NAFLD patients, with epidemiological studies linking low serum 25-hydroxyvitamin D levels to the NAFLD disease. Vitamin D may influence NAFLD progression through multiple mechanisms, including modulating immune responses and inflammation^38,39^, enhancing insulin sensitivity to counteract metabolic syndrome features^40^, and regulating lipid metabolism to reduce intrahepatic fat accumulation^41^. Vitamin E, due to its antioxidant properties, helps mitigate oxidative stress, a major contributor to NAFLD progression into NASH. Additionally, recent research suggests that vitamin K may support liver health by regulating lipid metabolism and inflammation. Emerging evidence indicates that vitamin K supplementation could enhance liver function and reduce fibrosis in chronic liver diseases, including NAFLD^39^, however, data of this study revealed no relation between NAFLD and the dietary intake of vitamin k.

This research demonstrated significant negative correlations between the intake of vitamins B6 and B12 and the risk of developing NAFLD, indicating a beneficial effect. The intake of folic acid exhibited an inverse relationship solely among obese individuals with moderate NAFLD only, while vitamin C showed a similar association in those with mild NAFLD. These findings were in accordance with previous research showing that vitamins B6, B12 and folate are essential for liver metabolism and may help prevent fat accumulation and inflammation, thereby reducing the risk and progression of NAFLD^42^^43^. Therefore, maintaining adequate intake of these micronutrients may support liver health in obese individuals. Vitamin C, a powerful antioxidant, plays a protective role against NAFLD by reducing oxidative stress and liver inflammation through neutralization of reactive oxygen species (ROS). Animal studies show that vitamin C supplementation significantly decreases liver steatosis and oxidative damage, especially in models fed a choline-deficient diet^44^. Epidemiological research supports these findings, revealing that higher dietary intake of vitamin C is linked to improved liver function and glucose metabolism. In Chinese adults, increased vitamin C consumption was associated with lower plasma ferritin and HbA1c levels, suggesting better metabolic regulation^45^. Vitamin C may also aid in lipid homeostasis, offering protection against NAFLD, especially in those with poor diets. Additionally, a non-linear relationship has been observed between serum vitamin C levels and NAFLD risk, with levels below 0.92 mg/dL significantly increasing the likelihood of disease^46^.

Finally, this study demonstrated that obese women with moderate NAFLD tended to have higher caloric intake, particularly from fats and carbohydrates, lower fiber consumption, and a greater prevalence of sedentary behavior compared with other groups. These patterns were accompanied by higher body mass index and waist circumference, as well as more frequent hyperglycemia and dyslipidemia. Such findings are consistent with previous reports linking lifestyle and metabolic factors to NAFLD^47^. Our results indicated that modifiable lifestyle factors, including physical inactivity, general obesity and high intake of calorie-dense foods, were associated with NAFLD, while central obesity was observed in all affected participants. Among metabolic comorbidities, only hypertension showed a significant association. Notably, a considerable proportion of participants with NAFLD were asymptomatic, supporting the potential value of early screening and lifestyle-based preventive strategies.

## Conclusion

This study demonstrated a high prevalence of NAFLD among obese middle-aged Egyptian women and identified significant associations between NAFLD severity, central obesity and dietary patterns. Women with NAFLD more frequently reported excessive caloric intake, higher consumption of refined carbohydrates and unhealthy fats and lower fiber and micronutrient intakes. These findings suggest that nutritional habits may represent modifiable risk factors of NAFLD in this population. The results provide important groundwork for future longitudinal and interventional studies aimed at elucidating dietary risk pathways and developing targeted nutritional strategies for NAFLD prevention and management.

## Strengths and limitations

This study offers focused evidence on NAFLD prevalence and severity among middle-aged obese Egyptian women, a population that remains underrepresented in the literature. Its main strength lies in the comprehensive assessment combining dietary analysis, anthropometric measurements, ultrasonography, and biochemical markers, allowing an integrated evaluation of nutritional and lifestyle factors associated with NAFLD. The detailed assessment of macro- and micronutrient intake further highlights potentially modifiable dietary risks.

However, the study is limited by recruitment from a weight-loss program and a relatively small sample size, which may restrict generalizability. Analyses were limited to unadjusted univariate and bivariate methods without correction for multiple comparisons. Dietary intake and physical activity were self-reported which may subject to recall bias. Additionally, socioeconomic factors, medication use, and objective physical activity measures were not fully assessed. Larger, population-based studies are warranted.

## Data Availability

The data supporting the findings of this study are available from the corresponding author upon reasonable request, after taking the permission of our institute “National Research Centre”.

## Abbreviations

ALT: Alanine aminotransferase
AST: Aspartate aminotransferase
BMI: Body mass index
DBP: Diastolic blood pressure
FBG: Fasting blood glucose
GGT: Gamma-glutamyl transferase
HDL-C: High-density lipoprotein cholesterol
MASLD: Metabolic-associated fatty liver disease
MENA: Middle East and North Africa
MUFAs: Monounsaturated fatty acids
NAFLD: Nonalcoholic fatty liver disease
NASH: Nonalcoholic steatohepatitis
NRC: National Research Centre
PUFAs: Polyunsaturated fatty acids
RDA: Recommended daily allowance
SBP: Systolic blood pressure
SFAs: Saturated fatty acids
T. bilirubin: Total bilirubin
T. protein: Total protein
T2DM: Type 2 diabetes mellitus
TC: Total cholesterol
TG: Triglyceride
UFAs: Polyunsaturated fatty acids
WC: Waist circumference
WHtR: Waist to height ratio
